# All-Polymer Solar Cells Sequentially Solution Processed from Hydrocarbon Solvent with a Thick Active Layer

**DOI:** 10.3390/polym15163462

**Published:** 2023-08-18

**Authors:** Yajie Wang, Chaoyue Zhao, Ziqi Cai, Lihong Wang, Liangxiang Zhu, Hui Huang, Guoping Zhang, Peng You, Chen Xie, Yaping Wang, Qing Bai, Tao Yang, Shunpu Li, Guangye Zhang

**Affiliations:** 1 College of New Materials and New Energies, Shenzhen Technology University, Shenzhen 518118, China202003020103@stumail.sztu.edu.cn (G.Z.);; 2 Julong College, Shenzhen Technology University, Shenzhen 518118, China; 202100501059@stumail.sztu.edu.cn (Z.C.);

**Keywords:** all-polymer solar cells, sequential processing, hydrocarbon solvent processing, thick-film active layer

## Abstract

Organic solar cells (OSCs) have gained increasing attention. Among the various directions in OSCs, all-polymer solar cells (all-PSCs) have emerged as a highly promising and currently active research area due to their excellent film formation properties, mechanical properties, and thermal stabilities. However, most of the high-efficiency all-PSCs are processed from chloroform with an active layer thickness of ~100 nm. In order to meet the requirements for industrialization, a thicker active layer processed from low-vapor pressure solvents (preferentially a hydrocarbon solvent) is strongly desired. Herein, we employ toluene (a hydrocarbon solvent with a much higher boiling point than chloroform) and a method known as sequential processing (SqP) to mitigate the rapid decline in efficiency with increasing film thickness. We show that SqP enables a more favorable vertical phase segregation that leads to less trap-assisted recombination and enhanced charge extraction and lifetime than blend-cast devices at higher film thicknesses.

## 1. Introduction

With the increasing emphasis on energy, organic solar cells (OSCs) have gained much more research attention [1,2,3,4,5]. OSCs, characterized by their light weight, flexibility [6], transparency, and low cost, have achieved power conversion efficiencies (PCEs) exceeding 19% [7,8,9,10]. Among different types of OSCs, all-polymer solar cells (all-PSCs), whose active layer is composed of polymer donors and polymer acceptors, have developed rapidly in recent years [11,12,13,14]. All-PSCs exhibit enhanced film-formation properties due to their long polymer chains compared to small molecules, improved thermal stability and photostability, and excellent mechanical properties [15,16,17]. These advantages make them great candidates for large-scale production and potential commercialization [18,19,20]. Particularly, the emergence of polymerized small molecule acceptors has pushed the efficiency of all-PSCs to over 18%, approaching the efficiency of non-fullerene small molecule OSC systems [21,22,23,24]. However, there are still issues to be solved in order to facilitate the progress of large-scale production for all-PSCs.

First, similar to other types of OSCs, the thickness of the optimized all-PSC device is typically around 100 nm [25,26,27,28]. However, for the industrialization of OSCs, the fabrication of thicker active layers is desired for several reasons: thick active layers can be easily obtained through various fabrication methods, they effectively reduce the device’s short-circuit rate, and they can harvest more photons [29,30,31,32,33,34,35,36]. Second, chloroform is the mainstream solvent for active layer processing of all-PSCs with state-of-the-art efficiency. Nevertheless, chloroform is not the optimal choice for industrialization due to its low boiling point and carcinogenic properties [18,19,20]. The morphology of chloroform-processed active layers is typically in a nonequilibrium state that could reduce the overall stability. However, employing alternative non-halogen solvents generally leads to a decrease in device efficiency. This is primarily attributed to the insufficient solubility of the active layer in non-halogen solvents [37,38]. To address these issues, the core objective of this work is to find a means to mitigate the rapid decline in efficiency with increasing active layer thickness in high-efficiency all-PSCs using non-halogen solvents.

In this study, we employed the sequential processing (SqP) technique to fabricate the active layer, which differs from the traditional blend-casting (BC) technique (Figure 2a). This approach allows better control over the vertical morphology of the active layer [39]. We successfully prepared all-PSCs with thicknesses up to 310 nm for the organic medium using toluene (Tol) as the processing solvent for the active layer. At high film thicknesses, SqP provides a PCE of 14.2% at a 310 nm thickness, which is much higher than that of BC (12.4%). We systematically compared the film properties and device performance between the SqP and BC methods at three representative active layer thicknesses: 75 nm (with the highest efficiency of 16.1%), 185 nm, and 310 nm. Our results demonstrate that SqP devices not only outperform BC devices in the case of the high-efficiency (75 nm) active layer but also maintain the core parameters of the devices more effectively under thick-film conditions. Through film-morphology investigation, we show that the SqP method can significantly improve the vertical phase segregation of the active layer, particularly at high film thicknesses. This contributes to improved charge extraction, an enhanced transient photovoltage decay constant, and reduced trap-assisted recombination, which provides the SqP devices with much smaller fill factor decreases at high active-layer thicknesses. Overall, our results manifest the effectiveness of the SqP method in making efficient all-PSC devices, reducing the efficiency drop at high film thicknesses, and replacing the use of chloroform with a high-boiling point hydrocarbon solvent. Our result is among the highest PCEs for thick-film all-PSCs processed from non-halogen solvents.

## 2. Experiments

### 2.1. Materials

The donor polymer, PM6, was purchased from Solarmer Material Inc. in Beijing, China. The acceptor polymer, PYF-T-*o*, and the electron transport materials, PNDIT-F3N, were purchased from eFlexPV Limited in Guangdong, China, known for their excellence in providing cutting-edge materials. The hole transport material, PEDOT: PSS (Clevios P VP 4083), was procured from Heraeus Inc. in Hanau, Germany. Moreover, all other reagents and chemicals were purchased from Sigma Aldrich (Saint Louis, MO, USA) or Aladdin (Delaware, IA, USA) and used as received.

Full names for some of the materials:

PM6: Poly[(2,6-(4,8-bis(5-(2-ethylhexyl-3-fluoro)thiophen-2-yl)-benzo [1,2-b:4,5-b’]dithiophene))-alt-(5,5-(1′,3′-di-2-thienyl-5′,7′-bis(2-ethylhexyl)benzo[1′,2′-c:4′,5′-c’]dithiophene-4,8-dione)]

PEDOT: PSS: poly(3,4-ethylenedioxythiophene): polystyrene sulfonate

PNDIT-F3N: y[(9,9-bis(3′-(N,N-dimethylamino)pro-pyl)2,7-fluorene)-alt-5,5′-bis(2,2′-thiophene)-2,6-naphtha-lene1,4,5,8-tetracabox-ylic-N,N′-di(2-ethylhexyl)imide].

### 2.2. Device Fabrication

The patterned indium tin oxide (ITO)-coated glass substrates were precleaned using detergent and then sonicated with deionized water, acetone, and isopropanol for 30 min. Then, the cleaned ITO substrates were dried in an oven overnight. Additionally, the ITO glass substrates were treated with UV–ozone for 15 min before PEDOT: PSS was spin coated on top at 5000 rounds per minute (rpm) for 15 s and then annealed at 150 °C on a hotplate for 10 min in atmosphere. The solar cells were fabricated in a conventional device configuration of ITO (50 nm)/ETL (PEDOT: PSS) (25~30 nm)/active layer (PM6: PYF-T-*o*)/HTL (PNDIT-F3N) (5~10 nm)/Ag (100 nm). Next, active layers with thicknesses ranging from 75 nm to 310 nm, which are shown in Table 1, were prepared. In this study, for all the devices, the active layers were formed by spin coating a toluene solution of the active layer material(s) in a nitrogen-filled glove box.

Active layer fabrication: For BC devices, the total donor/acceptor (D/A) weight ratio was kept at 1:1.2 in the PM6:PYF-T-*o* all-polymer blend solutions. Specifically, the concentrations of the PM6:PYF-T-*o* all-polymer blend solutions were 6 mg mL^−1^, 8 mg mL^−1^, and 10 mg mL^−1^ for the 75 nm-, 185 nm-, and 310 nm-thick active layer devices, respectively. Additionally, the 1-chloronaphthalene (CN) used as the solvent additive was added to the mixed solutions with 1%, 2%, and 2% vol ratios corresponding to the 75 nm, 185 nm, and 310 nm BC devices, respectively. As for the SqP devices with an active layer thickness of 75 nm, the donor (PM6) was dissolved in toluene (the concentration of the donor was 8 mg mL^−1^), and the acceptor (PYF-T-*o*) was also dissolved in toluene (the concentration of the acceptor was 12 mg mL^−1^) with 2% vol CN as the solvent additive. Turning to the 185 nm- and 310 nm-thick SqP devices, the concentrations of the donor solutions were increased to 12 mg mL^−1^ and 16 mg mL^−1^, with 2% and 6% (vol%) CN separately as the solvent additive, while 4% and 6% (vol%) CN were added into the 16 mg mL^−1^ and 20 mg mL^−1^ of the acceptor solutions, respectively. Both the donor and acceptor solutions used in the SqP devices and the all-polymer blend solutions in the BC devices were stirred for 30 min at 60 °C for intensive mixing in a nitrogen-filled glove box. To be specific, the donor solution was spin coated at 4000 rpm for 25 s onto the PEDOT: PSS films. It is noticeable that the acceptor solution was immediately spin coated using the same setup as the donor solution onto the donor films. The active layer was successfully prepared using the SqP method thus far. The following procedure was thermal annealing at 100 °C for 5 min.

Subsequently, for all types of devices that had been made before, methanol with a 0.5% vol acetic acid blend solution of PNDIT-F3N at a concentration of 0.5 mg mL^−1^ was spin coated onto the active layer. The spin-coating condition of PNDIT-F3N was 2000 rpm for 25 s. Finally, at a vacuum level of 2 × 10^−4^ Pa, approximately 100 nm-thick Ag was deposited using thermal evaporation through a shadow mask as the top electrode. In addition, the intact encapsulation was carefully carried out for some of the characterizations.

### 2.3. Device Characterization

Central to the characterization of all the devices are the current density–voltage (*J-V*) curves, which provide core parameters and crucial insights into the devices’ electrical behavior. These measurements are conducted under simulated AM 1.5 G irradiation, replicating solar illumination with a light intensity of 100 mW cm^−2^. To ensure accuracy in light intensity and spectral matching, a standard silicon reference cell with a KG5 filter was utilized. The definition of the standard device area assumes paramount importance in the characterization process. To this end, an optical microscope, specifically the Olympus BX51, was employed to accurately delineate the device area (7.2 mm^2^). The characterizations were measured in a glove box filled with high-purity nitrogen, and it was ensured that the oxygen and water contents remained below 0.1 ppm. The measurements of external quantum efficiencies (EQEs) were measured using an Enlitech QE-S EQE system equipped with a standard silicon diode. The monochromatic light utilized for the EQE measurements emanates from the Enlitech 300 W lamp source.

### 2.4. Analysis and Characterization

**Film-depth-dependent light absorption spectroscopy (FLAS) and composition distribution:**

Film-depth-dependent light absorption spectra were acquired using an in situ spectrometer, the renowned PU100, crafted by Shaanxi Puguang Weishi Co., Ltd. (Xi’an, China). Complementing this advanced instrument was a soft plasma ion source, generating soft ions with a 100 W power supply and an input oxygen pressure of approximately 10 Pa. What set this measurement apart was its ability to etch the film surface incrementally, all while preserving the underlying materials intact. This remarkable feat was accomplished thanks to the real-time monitoring capabilities of the spectrometer, granting us insights into the evolving dynamics within the film. The application of Beer–Lambert’s law served as the guiding principle. This methodical approach paved the way for the extraction of film-depth-dependent absorption spectra, unlocking information about the film’s light interactions at various depths. The sublayer absorption with pure components was fitted, unraveling the composition distribution along the film-depth direction. By inputting the sublayer absorption spectra into a modified optical transfer-matrix approach, the exciton generation contour was masterfully simulated, a crucial optical characteristic of the film.

**Transient photovoltage (TPV) measurements:**

In the TPV measurements, the devices were continuously irradiated using a light source, which is enabled with a focused quartz–tungsten–halogen lamp with an intensity similar to working devices, i.e., that promotes a steady and constant *V*_OC_. It is clear that the device voltage was close to the *V*_OC_ under solar illumination conditions. While photoexcitations were generated with 8 ns pulses from a laser system (Oriental Spectra, NLD520, Hyderabad, India), the wavelength for excitation was tuned to 518 nm with a spectral width of 3 nm. Meanwhile, the devices were connected to a digital oscilloscope, which can register changes in voltage over time and eventually acquire the TPV signals.

**Transient photocurrent (TPC) measurements:**

Turning to the TPC measurements, they were measured similarly under the same conditions as the TPV measurements, including the excitation wavelength, so that the basic setup of the TPC experiment was close to that of the TPV. However, it is an exception that TPC decays were held in short-circuit conditions without background light bias.

**Surface stylus profilers and thickness measurements:**

A stylus-based surface profiler purchased from KLA Instruments uses a stylus for tracing surface contours to acquire height and roughness information. In this study, height measurements were primarily used for the thickness of the devices. When the sensor assembly monitors the stylus movement as the sample is scanned, the stylus profiler measures surface topography with high sensitivity, utilizing sophisticated hardware and software. To be specific, after the movements of the stylus, there are small peaks and valleys on the surface that reflect the various surface contours with many clear steps. Sequentially, the electrical signal output by the sensor about the surface contours was calculated and eventually exhibited in the software.

## 3. Results and Discussion

**Figure 1:**
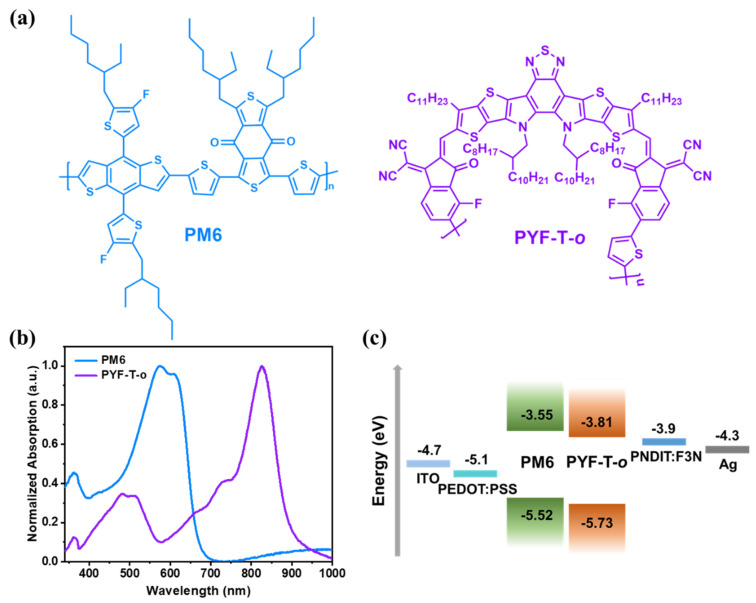
(**a**) Chemical structures of the donor, PM6, and the acceptor, PYF-T-*o*; (**b**) UV–vis absorption spectra of pure PM6 film and PYF-T-*o* film; (**c**) energy levels of the materials.

The chemical structures of the donor and acceptor materials, PM6 and PYF-T-*o*, are depicted in Figure 1a, together with the UV–vis absorption spectra of the thin films of the pure materials spin coated from toluene, depicted in Figure 1b and Figure S1. The absorption spectra of PM6 predominantly occur in the range of 500 nm to 700 nm, while PYF-T-*o* exhibits two distinct absorption regions, namely, 750–900 nm and 450–550 nm. The complementary absorption of the donor and acceptor enables higher photon absorption under identical conditions. The energy level distribution was as follows: highest occupied molecular orbit (HOMO) and lowest unoccupied molecular orbit (LUMO) of all materials applied in this research (Figure 1c). Of particular interest are the energy levels of the active layer materials: PM6 exhibits HOMO and LUMO levels at −5.52 eV and −3.55 eV, respectively, while PYF-T-*o* possesses HOMO and LUMO levels at −5.73 eV and −3.81 eV, respectively.

**Figure 2:**
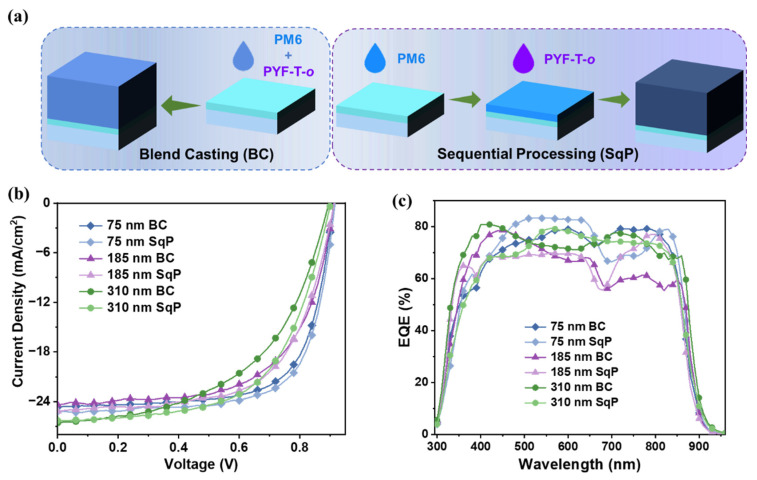
(**a**) Schematic diagram of the procedure for fabricating the active layer using BC and SqP; (**b**) current density–voltage (*J–V*) curves of the different devices; (**c**) external quantum efficiency (EQE) spectra of the different devices.

Figure 2a compares the different active layer deposition processes, blend casting (BC) and sequential processing (SqP), in an abridged general view. The device performance comparison is shown in Figure 2b, with the current density versus voltage (*J–V)* characteristics, and in Table 1. The six devices prepared by the same materials, PM6 and PYF-T-*o*, as the active layer, were named after different active layer thicknesses and various methods, like 75 nm BC and 75 nm SqP. The 75 nm BC devices displayed a PCE of 15.6%, with a *V*_OC_ of 0.911 V, a *J*_SC_ of 24.6 mA cm^−2^, and an FF of 69.4%, while the optimal performance with a PCE of 16.1%, a *V*_OC_ of 0.915 V, a *J*_SC_ of 25.3 mA cm^−2^, and an FF of 69.9% was obtained with an active layer thickness of 75 nm prepared using the SqP method. With the increase in active layer thickness, the *V*_OC_ changed slightly, while the FF declined dramatically. When the film thickness reached up to 185 nm, the *J*_SC_ started to descend, but high performance over 14% was still maintained using the SqP method. With decreasing FFs in the thick-film devices, there was a PCE containing at least more than 12% in the 310 nm devices used with the BC and SqP methods. In contrast, the thick SqP devices maintained notable advantages in FF, which were higher than the devices fabricated with the BC method. Therefore, SqP resulted in the highest PCE of 14.2% in the 310 nm active-layer devices. Then, we conducted the EQE measurement, as is shown in Figure 1c. From the EQE curves, we found that the current density obtained from the EQE integration was consistent with the *J*_SC_ obtained from the *J–V* test (Figure 2b and Table S1).

**Figure 3:**
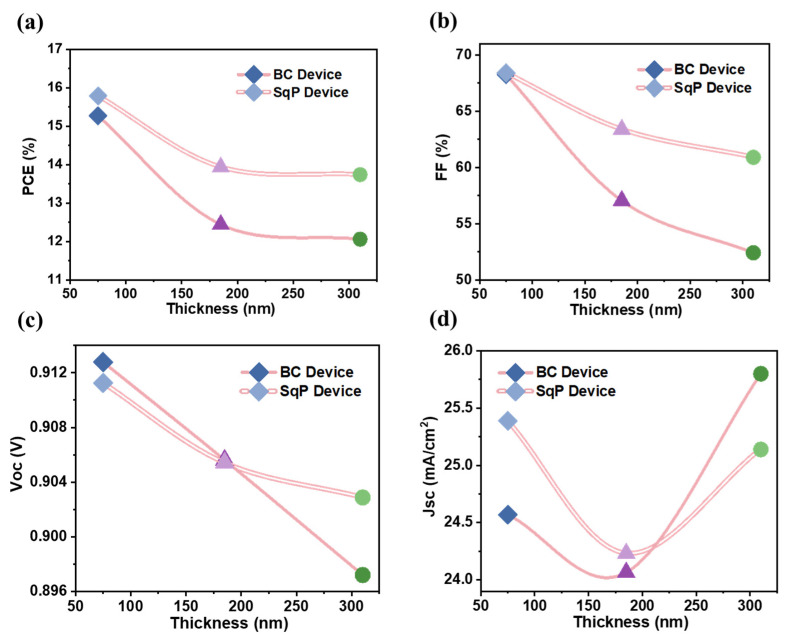
Plot of trends of PCEs (**a**), FF (**b**), *V_OC_* (**c**), and *Jsc* (**d**) with respect to active layer thickness.

In order to more intuitively display the state of change in the performance parameters of the device with the thickness of the active layer, we conducted a series of characterizations. As shown in Figure 3, we plotted the average data measured by the devices with different thicknesses using different methods (including BC and SqP) and obtained four comparison diagrams of four core parameters. It is obvious that PCE and FF declined dramatically when the thickness of the active layer increased, as shown in Figure 3a,b. However, the devices fabricated with the SqP method always had better performance than the BC method. It is interesting that the PCE (averaged value) fell from 15.8% to 13.9% in the SqP method as the thickness of the active layer rose from 75 nm to 185 nm, which is a noticeable change, but when it increased from 185 nm to 310 nm, the change in PCE was minimal, which was eventually at 13.7% (Figure 3a). As for the BC method, the PCE had a more remarkable decrease, falling from 15.6% to 12.4%, and finally dropped at 12.1% (Figure 3a). Turning to *V*_OC_ displayed in Figure 3c, it also maintained the same downward trend but changed less than the other parameters, which changed from 0.911 V to 0.905 V and finally fell at 0.902 V (compared with the average data) using the SqP method. It is interesting that *J*_SC_ had a totally different trend, and it seemed to fluctuate during the change of thicknesses in the active layer, which is shown in Figure 3d.

**Figure 4:**
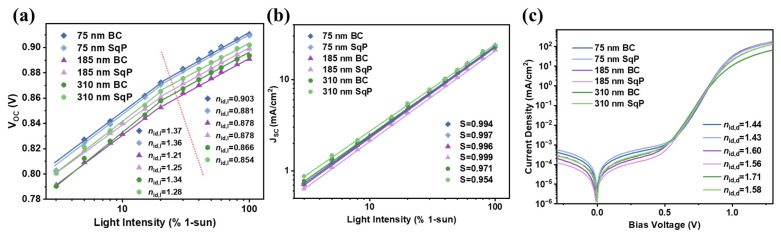
(**a**) *V*_OC_ versus light intensity of devices; (**b**) *J*_SC_ versus light intensity of devices; (**c**) dark *J*–*V* curves of devices.

To better understand device performance, light intensity studies were first performed to analyze the charge recombination. By fitting the *V*_OC_ versus ln(*I*) result in Figure 4a, ideality factors (*n*_id_) can be calculated, which have been depicted to relate to trap-assisted recombination based on the diode theory. The ideality factors (*n*_id,l_) under different light intensities can be calculated with $n_{id,l}=\frac{q}{kT}\frac{\partial V_{oc}}{\partial \ln\left(I\right)}$, where *I* is the light intensity, *q* is the elementary charge, *k* is the Boltzmann constant, and *T* is the Kelvin temperature. Meanwhile, the change in *J*_SC_ with respect to light intensity is plotted in Figure 4b on a logarithmic scale. The slope (*S*) obtained from linear fittings is listed in Table 1, which has been adopted in the field to indicate bimolecular recombination. The *S* values for the BC and SqP devices are 0.994 and 0.997 at the 75 nm active layer thickness, respectively. It is shown that the SqP devices had less bimolecular recombination. When it comes to the 185 nm active layer devices, the *S* values for the two methods are 0.996 and 0.999, while the *S* values for the 310 nm active layer devices decreased significantly compared with the thinner film devices. At low light intensities, Figure 4a compares the *n*_id,l_ of the different devices (Figure 4a): the SqP device has similar or even less trap-assisted recombination relative to the BC device. To further verify the ideality factor between SqP and BC, we fitted the exponential region of the dark *J–V* curves, where the dark ideality factor (*n*_id,d_) can be calculated with $n_{id,d}=\frac{q}{kT}\frac{\partial V}{\partial J}$, where *q*, *k*, *T*, *V*, and *J* are the fundamental charge, Boltzmann constant, bias voltage, and dark current density, respectively. The dark *J–V* curves are plotted in Figure 4c, and the *n*_id,d_ values are calculated using the slope of the linear region, and at the same time, they are also listed in Table 1. From the fitting, the *n_id,d_* of the BC and SqP devices shared great similarity, with each rising when the thickness of the active layer increased. To be specific, the *n*_id,d_ of BC with the three thicknesses of the active layer are 1.44, 1.60, and 1.71, respectively, while the values for SqP are 1.43, 1.56, and 1.58, respectively. The trends of the results for *n*_id,d_, *S*, and *n*_id,l_ are basically the same, indicating that the SqP devices have some advantages in reducing carrier recombination.

**Figure 5:**
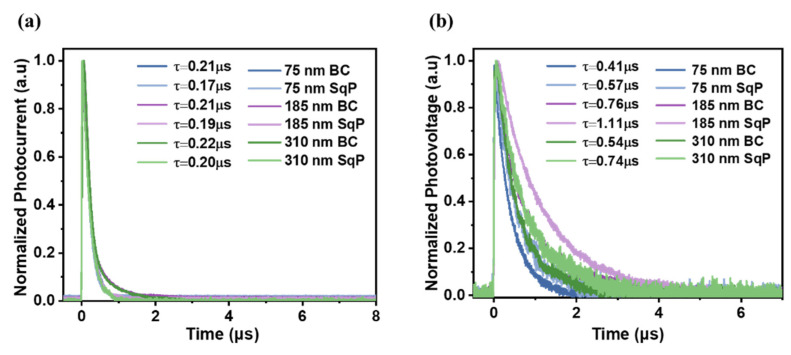
(**a**) Transient photocurrent (TPC) and (**b**) Transient photovoltage (TPV) based on different devices.

To further study the carrier lifetime and carrier extraction time, we performed transient photocurrent (TPC) and transient photovoltage (TPV) measurements. The normalized TPC and TPV curves are shown in Figure 5, and the mean values are listed in Table 1. As exhibited in Figure 5a, from the TPC, the carriers of the 75 nm SqP devices are extracted faster (0.17 μs) than the 75 nm BC devices (0.21 μs). When the thickness of the active layer rises to 185 nm, the values of the TPC of the BC and SqP methods are 0.21 μs and 0.19 μs, respectively, which rise to 0.22 μs and 0.20 μs at the 310 nm thick-film device, respectively. Meanwhile, this indicates that the method of SqP can accelerate the carrier extraction rate in the thick-film device. As for the values of TPV, the carrier lifetime (*τ*) of the 75 nm SqP device is 0.57 μs, which exceeds that of the BC device (*τ* = 0.41 μs). It is obvious that the gap between the two methods in the TPV values for the 185 nm devices is enlarged, at 0.76 μs and 1.11 μs in the BC and SqP devices, respectively. When it comes to the 310 nm devices, the carrier lifetime of the BC device is 0.54 μs, and that of the SqP device is 0.74 μs. Figure 5b illustrates that the SqP method not only prolongs the charge carrier lifetime in conventional devices with a normal thickness of the active layer but also plays a vital role in thick-film devices.

**Figure 6:**
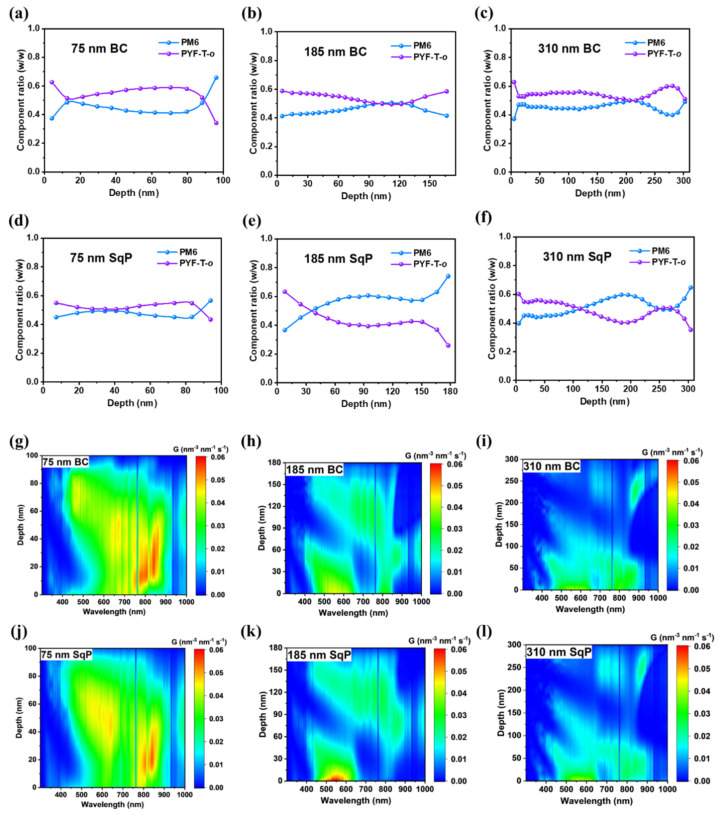
(**a**–**f**) Composition ratio across the vertical direction of the active layer film calculated from the results of FLAS; (**g**–**l**) exciton generation map across the vertical direction of the active layer film as a function of wavelength. ETL and HTL stand for electron transport layer and hole transport layer, respectively.

After analyzing the device recombination and optoelectronic properties, we turned to study the morphology of different thickness devices using various methods. We employed film-depth-dependent light absorption spectroscopy (FLAS) to study the vertical phase segregation of the active layer. This technique utilizes low-pressure plasma to incrementally etch the film from the top surface (electron transport layer/active layer interface) to the bottom of the active layer (hole transport layer interface) (Figure S2). The sublayer spectra were obtained by subtracting the spectra of adjacent layers and subsequently shifting (Figure S3). Based on the transfer matrix model in the measurement software, the vertical composition distribution of the active layer (Figure 6a–f), vertical exciton distribution maps (Figure 6g–l), and vertical exciton generation rate maps (Figure S4) were determined. Of particular interest is the vertical composition distribution map (Figure 6a–f), which reveals distinct distributions for different thicknesses. In the 185 nm and 310 nm devices, the use of the SqP method allows for greater PM6 enrichment near the HTL, thereby increasing the hole transmission probability to a certain extent. Regarding the vertical exciton distribution maps (Figure 6g–l), in the 75 nm device, excitons are primarily generated within the first 60 nm depth, while in the 185 nm device, excitons are generated throughout the entire active layer, and in the 310 nm device, excitons are primarily generated within the first 200 nm. It is noteworthy that there is minimal exciton generation in the subsequent 110 nm, indicating that the hole needs to migrate a longer distance compared to the electron, making hole charge collection more difficult in thicker film devices. However, the SqP method effectively addresses this issue by increasing the proportion of the donor near the HTL, thereby increasing the probability of hole charge collection. This phenomenon becomes more pronounced as the film thickness increases.

## 4. Discussions on Film Thickness

In a sequentially solution-processed (SqP) active layer, the overall thickness, along with the donor to acceptor ratio, is particularly important for device performance and for direct comparison with the corresponding blend-casting (BC) devices. In particular, when the active layer is made using the spin-coating technique, the underlayer is swollen by the solution of the top layer. The degree of swelling must be between total dissolving and not-wetting in order to form a working BHJ [40,41,42,43]. In our recent report on an all-PSC system, we found that the deposition of the top-layer solution made the underlayer undergo a solid–liquid–solid phase transition, but the time it went into the liquid phase was rather short compared to the BC method, which allows much more time for both the donor and acceptor to be in solution together [39]. Unlike blade-coating or slot-die coating, during the spin-coating process, there may be a fraction of the underlayer that is washed away by the top-layer solution. This effect could be minimal in the all-PSC system, as the underlayer is formed with polymers with high-molecular weights, such as PM6 (the viscosity is high).

Therefore, the total thickness of the active layer in the SqP method depends on multiple factors: the concentration and coating conditions of the underlayer, the concentration and coating conditions of the top-layer solution, the degree of swelling of the underlayer by the top solution, and the crystallinity of the materials. It is thus difficult to predict the overall thickness and the donor/acceptor ratio of the resulting active layer just from the concentration of each solution or their spin-coating speed/time.

To make the comparison between SqP and BC fair, what we did in this work was fix the thickness of the total active layer while optimizing the device efficiency at each thickness. For instance, we prepared the SqP film from different spin speeds and solution concentrations, but we only chose those films that had identical film thicknesses to the BC to make sure all the comparisons were made at the same film thickness. Meanwhile, although the donor/acceptor ratio could be slightly different due to differences in morphology (as shown in the UV–vis absorption profiles, also pasted below), the PCEs we report at each thickness are the optimized ones under the thickness. Overall, we compare the optimized BC and SqP devices that have the same active layer thickness. In such comparisons, the solvent effect is already included.

## 5. Conclusions

In conclusion, by employing toluene as a solvent substitute for the traditional chloroform in active-layer processing, comparable efficiencies were achieved using the SqP technique, as previously reported. Moreover, the SqP devices exhibited improved thickness tolerance, maintaining an efficiency of 14.2%, even at a thickness of 310 nm (compared to 12.4% for BC at 310 nm). Carrier analysis of the devices with varying thicknesses revealed the significant advantages of the SqP devices at higher thicknesses. Furthermore, in the thick-film devices, SqP resulted in a higher proportion of the donor near the HTL compared to the acceptor, leading to an increased exciton separation probability and consequently higher device performance.

## Figures and Tables

**Table 1:** *J-V* characteristics and other device parameters for PM6, PYF-T-*o*-based all-PCSs processed from different methods, measured under AM 1.5 G illumination at 100 mW cm^−2^.

Thickness [nm]	Active Layer	*V*oc [V]	*J*sc [mA/cm^2^]	FF	PCE * [%]	TPC [μs]	TPV [μs]	*S*	*n*_id,d_
75	BC	0.913 ± 0.003 (0.911)	24.6 ± 0.3 (24.6)	0.682 ± 0.012 (0.694)	15.3 ± 0.2 (15.6)	0.204 ± 0.017	0.410 ± 0.007	0.994	1.44
	SqP	0.911 ± 0.003 (0.915)	25.4 ± 0.5 (25.3)	0.684 ± 0.014 (0.699)	15.8 ± 0.1 (16.1)	0.176 ± 0.003	0.525 ± 0.039	0.997	1.43
185	BC	0.906 ± 0.06 (0.912)	24.1 ± 0.4 (24.2)	0.570 ± 0.024 (0.619)	12.4 ± 0.5 (13.7)	0.196 ± 0.010	0.746 ± 0.018	0.996	1.60
	SqP	0.905 ± 0.08 (0.913)	24.2 ± 0.6 (25.0)	0.634 ± 0.013 (0.624)	13.9 ± 0.2 (14.2)	0.190 ± 0.004	1.095 ± 0.014	0.999	1.56
310	BC	0.897 ± 0.02 (0.896)	25.8 ± 0.4 (26.5)	0.524 ± 0.006 (0.524)	12.1 ± 0.2 (12.4)	0.223 ± 0.003	0.544 ± 0.009	0.971	1.71
	SqP	0.902 ± 0.001 (0.902)	25.1 ± 0.6 (26.3)	0.609 ± 0.006 (0.600)	13.7 ± 0.3 (14.2)	0.196 ± 0.007	0.726 ± 0.016	0.954	1.58

* The standard deviations are based on measurements of 10 independent devices. The values in brackets are the parameters corresponding to the devices with the highest PCE of each method.

## Data Availability

Not applicable.

## Supplementary Materials

The following supporting information can be downloaded at: https://www.mdpi.com/article/10.3390/polym15163462/s1. Figure S1. UV-Vis absorption of the blend films. Figure S2. (a–f) Film-depth-dependent light absorption spectra (FLAS) of the active layer for BC and SqP blend films at each thickness. Figure S3. (a–f) Absorption of the sub-layer calculated from FLAS spectra. Figure S4. (a–f) Exciton generation map across the vertical direction of the active layer film as a function of wavelength. Table S1. The JSC measured from J-V test and the values of EQE-integrated current density for PM6/PYF-T-o based all-PCSs processed from different methods, measured under AM 1.5 G illumination at 100 mW cm^−2^. Table S2. BC device performance with active layers under different processing parameters, measured under AM 1.5 G illumination at 100 mW cm^−2^. Table S3. SqP device performance with active layers under different processing parameters, measured under AM 1.5 G illumination at 100 mW cm^−2^.
