# Supplementary material for: All-Polymer Solar Cells Sequentially Solution Processed from Hydrocarbon Solvent with a Thick Active Layer
Source: Polymers (Basel). 2023 Aug 18;15(16):3462. doi: 10.3390/polym15163462 (PMC10459458; doi:10.3390/polym15163462)
Supplement: Supplementary file 1 [file polymers-15-03462-s001.zip › polymers-2543580-supplementary.pdf]

Supporting information

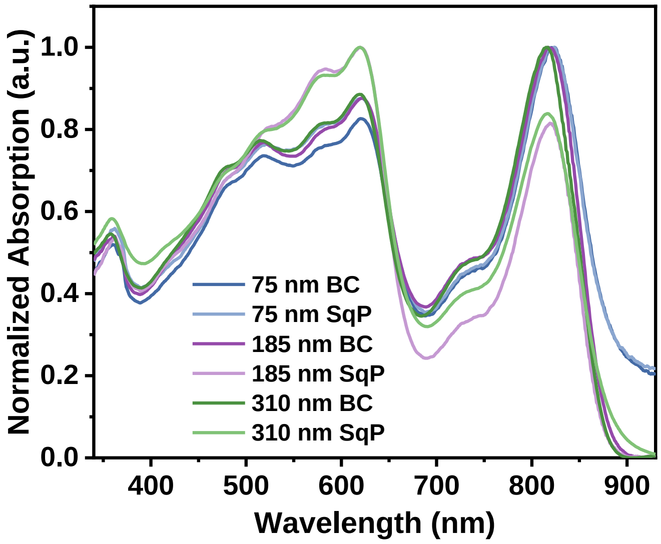

Figure S1. UV-Vis absorption of the blend films.

Table S1. The J_{SC} measured from J-V test and the values of EQE-integrated current density for PM6/PYF-T-*o* based all-PCSs processed from different methods, measured under AM 1.5 G illumination at 100 mW cm^{-2}.

Thickness [nm]	Active Layer	J_{SC} [mA cm^{-2}]	EQE Integrated Current Density [mA cm^{-2}]
75	BC	24.6±0.3 (24.6)	23.8
	SqP	25.4±0.5 (25.3)	24.3
185	BC	24.1±0.4 (24.2)	21.4
	SqP	24.2±0.6 (25.0)	21.7
310	BC	25.8±0.4 (26.5)	24.2
	SqP	25.1±0.6 (26.3)	24.9

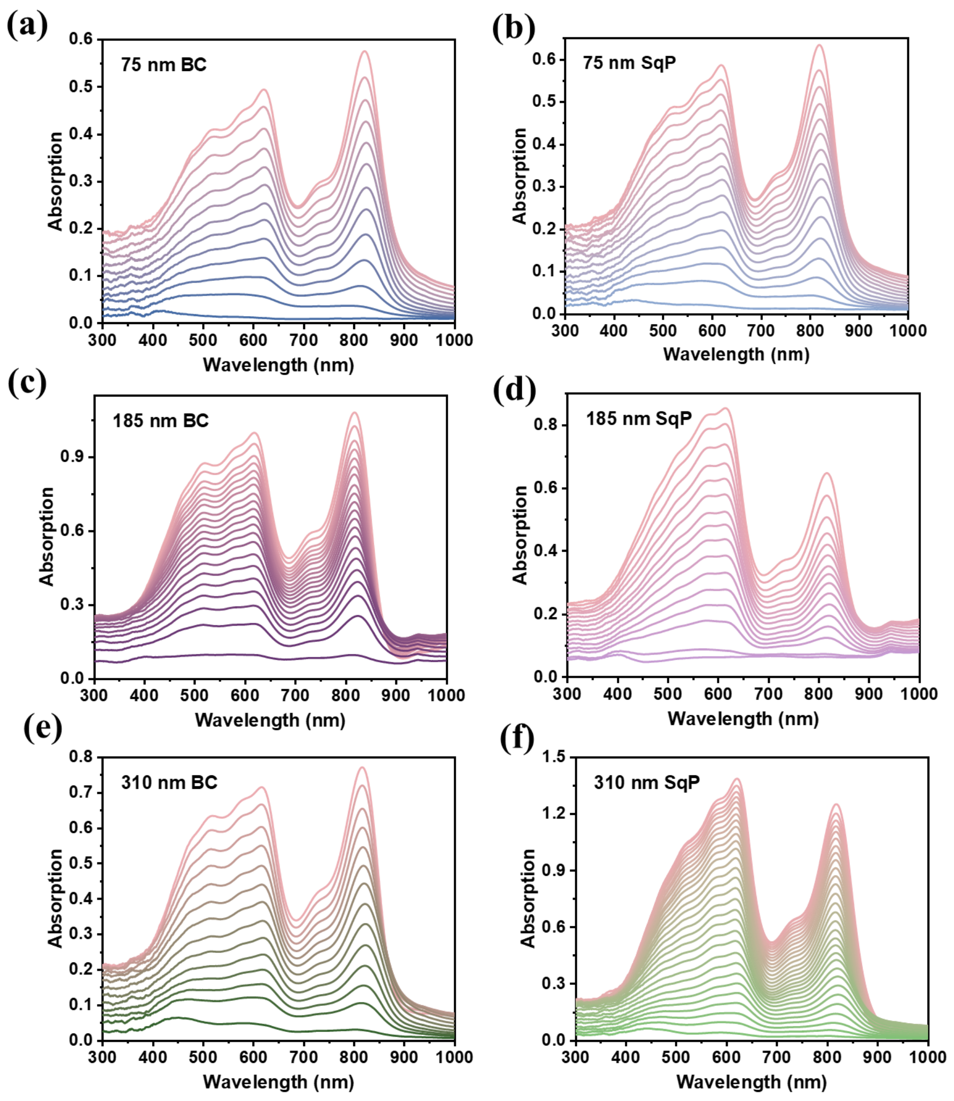

Figure S2. (**a**–**f**) Film-depth-dependent light absorption spectra (FLAS) of the active layer for BC and SqP blend films at each thickness.

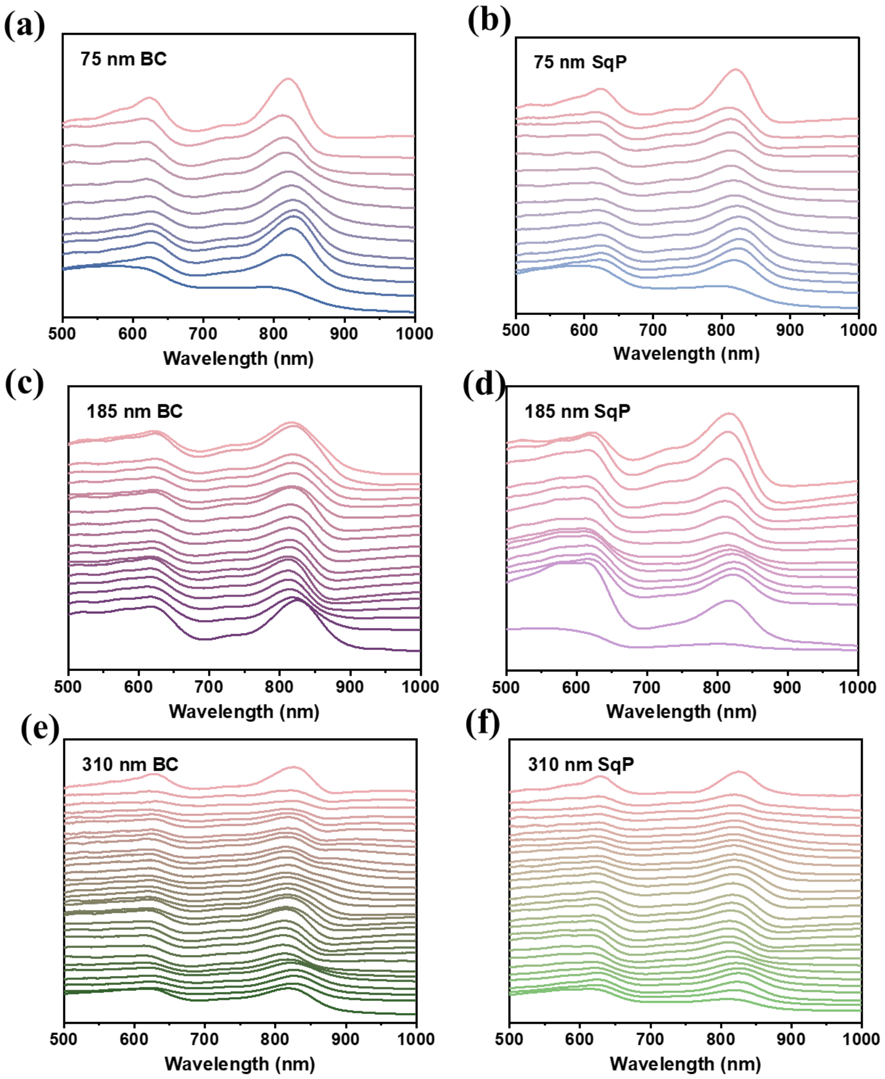

Figure S3. (**a–f**) Absorption of the sub-layer calculated from FLAS spectra.

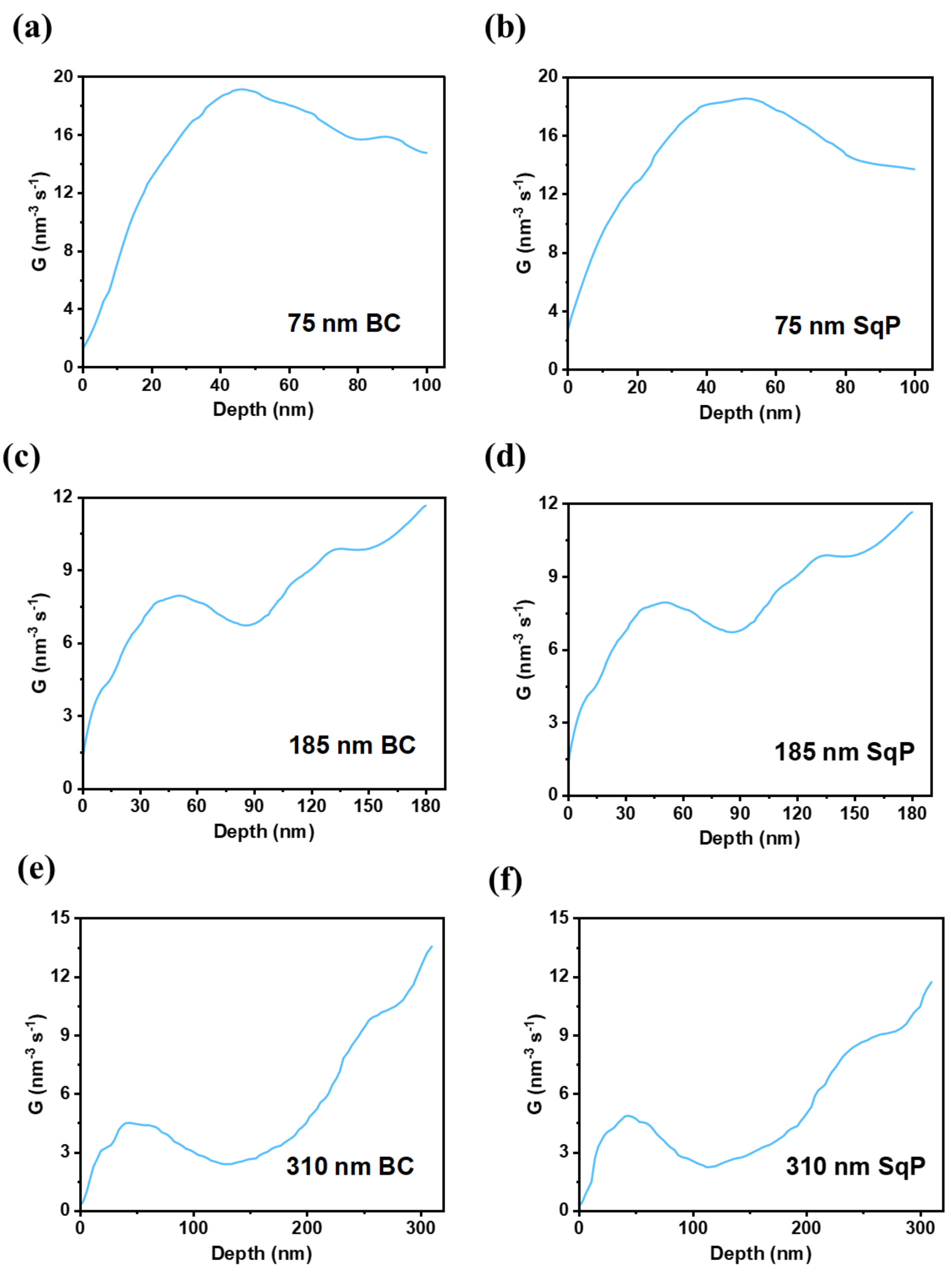

Figure S4. (**a**–**f**) Exciton generation map across the vertical direction of the active layer film as a function of wavelength.

Table S2. BC device performance with active layers under different processing parameters, measured under AM 1.5 G illumination at 100 mW cm^{-2}.

Devices	Speed [rpm]	Voc [V]	Jsc [mA/cm²]	FF	PCE [%]
**PM6: PYF-T-*o* (6 mg/mL + 1% CN)**	4000	0.914	24.5	0.690	15.4
	3000	0.914	24.8	0.684	15.5
	2000	0.901	25.0	0.603	13.6
**PM6: PYF-T-*o* (7 mg/mL + 1% CN)**	4500	0.906	24.5	0.588	13.0
	3500	0.904	23.9	0.555	11.9
**PM6: PYF-T-*o* (8 mg/mL + 2% CN)**	4000	0.907	24.2	0.550	12.1
**PM6: PYF-T-*o* (10 mg/mL + 1% CN)**	4000	0.895	21.8	0.441	8.6
	3000	0.896	21.9	0.401	7.9
	2000	0.890	20.9	0.394	7.2

**PM6: PYF-T-*o* (10 mg/mL + 2% CN)**	4000	0.895	25.9	0.526	12.2
	3000	0.892	24.6	0.478	10.5

Table S3. SqP device performance with active layers under different processing parameters, measured under AM 1.5 G illumination at 100 mW cm^{-2}.

**Devices**	**Speed [rpm]**	**Voc [V]**	**Jsc [mA/cm^2]**	**FF**	**PCE [%]**
**PM6 (8 mg/mL)<br>PYF-T-*o* (12 mg/mL + 2%CN)**	4000	0.922	24.5	0.707	15.7
	3000	0.912	25.9	0.680	16.1
	2000	0.913	24.3	0.637	14.0
**PM6 (12 mg/mL)<br>PYF-T-*o* (16 mg/mL + 2%CN)**	4000	0.917	23.6	0.606	13.1
	3000	0.913	22.1	0.564	11.4
	2000	0.904	18.1	0.554	9.1
**PM6 (12 mg/mL + 2%CN)<br>PYF-T-*o* (16 mg/mL + 4%CN)**	4000	0.914	23.8	0.636	13.8
**PM6 (16 mg/ml)<br>PYF-T-*o* (20 mg/ml+6%CN)**	4000	0.901	22.8	0.537	11.0
	3000	0.896	18.5	0.503	8.3
	2000	0.889	16.7	0.484	7.2
**PM6 (16 mg/mL: + 6%CN)<br>PYF-T-*o* (20 mg/mL + 6%CN)**	4000	0.901	25.1	0.604	13.6
